# “Net” value co-creation: The effect of interactions on consumer citizenship behavior in online travel communities

**DOI:** 10.3389/fpsyg.2022.991009

**Published:** 2022-08-15

**Authors:** Biyu Guan, Haiquan Chen, Yunhao Liu, Rui Liu, Ailing Wu

**Affiliations:** ^1^School of Management, Jinan University, Guangzhou, China; ^2^Business and Economics, University of Delaware, Newark, NJ, United States; ^3^Western Business School, Southwestern University of Finance and Economics, Chengdu, China; ^4^College of Tourism and Service Management, Nankai University, Tianjin, China

**Keywords:** consumer interaction, self-identity, perceived social support, community identification, citizenship behavior

## Abstract

Online traveling community is initiated by companies, but its survival is inextricably linked to consumer citizenship behavior (e.g., out-group recommendation, in-group helping, and inward response). The majority of researches have investigated consumer behavior of brand community such as consumer satisfaction, brand loyalty, and purchase intention. A few scholars try to explore consumer behaviors beyond the purchase, like participation, which was concerned as the value co-creation. However, the value co-creation of the community should depend on consumers’ citizenship behaviors instead of their pure participation. Therefore, this study empirically examines the effect of consumer interaction on consumer psychology and citizenship behaviors of the online travel community. The findings demonstrated that consumer interaction facilitated participants’ self-identity and their perceived social support, which enhanced their community identification and thus their citizenship behaviors. Furthermore, the motivation of participation plays a moderator in this process. Specifically, symbolic motivation moderates the relationship between consumer interaction and their self-identity, while utilitarian motivation moderates the effect of consumer interaction on their perceived social support. These findings contributed to the intervention of consumer citizenship behavior in online traveling community and provide insights into the management of the online travel community from the perspective of the value co-creation.

## Introduction

As the emergence of leisure and outdoor recreation, the online travel communities have been initiated by the companies to satisfy the travel enthusiast. The online travel communities are social platforms that enable tourists to search for and share related knowledge, information, and experience of travel (Lv et al., 2021). Most of the time, the online travel communities provide recommendations and supports for the tourists for free (Lv et al., 2021). These communities provide potential marketing and commercial value to the companies such as the maintenance of customer relationship and online advertisement (Lv et al., 2021; Xue et al., 2021). In the meantime, consumers can obtain benefits from the communities and support other members voluntarily through interactions in return, which contributes to the value co-creation of the online travel communities (Chang et al., 2020). Given these advantages, consumer interaction of the online travel communities has been the focus of many researches. Consumer interaction was defined as the extent to which consumers communicate and interact with each other in online communities (Casaló et al., 2017). Consumer interaction proved beneficial to both consumers and companies (Cao et al., 2021; Cheung et al., 2021; Liao et al., 2021). On the one hand, consumer interaction can foster information interaction and the community relationship (Cao et al., 2021; Cheung et al., 2021). On the other hand, this interaction can enhance consumers’ brand engagement, brand loyalty, and purchase intention (Liao et al., 2021). However, how do consumer interactions shape consumer behaviors beyond their behaviors toward the brand itself?

Extant research treated consumer interaction or participation as the value co-creation (Hsieh et al., 2022), but consumer interaction does not necessarily create value to the online travel communities. The intra-role consumer participation such as information searching and problem consulting did not bring about the actual creation of value. Only the extra-role consumer participation such as feedback, advocacy, and help creates actual value for the online travel communities (Yi et al., 2013; Lv et al., 2021). In this paper, we regard this extra-role participation as consumer citizenship behaviors, which means the behaviors that consumers provide their support to the communities voluntarily and without rewarding (Groth, 2005; Curth et al., 2014; Zhu et al., 2016). Regards to the antecedents of consumer citizenship behavior, there are several explanations such as the desire for online self-presentation (Wang et al., 2021), social capital, collective psychological ownership (Chi et al., 2022), and social wellbeing (Chou et al., 2021). Nevertheless, we propose that consumer interaction increases consumer citizenship behavior by strengthening members’ identification of community.

Consumer interactions help members build, affirm, and communicate their identities, which further help them obtain self-identity from the online travel communities (Yen et al., 2011; Xue et al., 2021). In the meantime, once consumers identify with their self-images through the recognition by other like-minded peers, they are prone to enhance their identifications with the community (Muñiz and O’Guinn, 2001). On the contrary, consumers can obtain not only the objective support (e.g., acquisition of information) but also the subjective support (e.g., respect from others) in the process of interactions (Yu et al., 2015; Xue et al., 2021). Consumer interactions can enhance members’ perception of social support and thus strengthen their identifications with members of the community (Chou et al., 2021). Furthermore, the identification of community can induce consumer to know more about the behavioral norms, goals, and culture of the community (Bhattacharya et al., 1995; Watts and Dodds, 2007). According to the social identity theory, consumers are more willing to make contributions to the community as their knowledge about the community increase (Bhattacharya et al., 1995; Dessart and Veloutsou, 2021). As a result, consumers are prone to conduct the citizenship behaviors such as recommendation, helping others, and providing feedback in the online travel communities (Hsu et al., 2015; Deng et al., 2021). Moreover, based on the theory of motivation, symbolic motivation can positively moderate the relationship between consumer interactions and their identification of the self-image, while utilitarian motivation positively moderates the effect of consumer interactions on their perception of social support (Dabbous and Barakat, 2020; Akram et al., 2021; Shukla and Rosendo-Rios, 2021; Iloranta and Komppula, 2022).

This paper has both theoretical and practical implications. It contributed to an understanding of the value co-creation of online community by clarifying its manifestation from consumers’ participation to consumer citizenship behavior. We argue that only the extra-role behavior derived from the participation can create additional values to the community. In addition, identification of community has proved to be the stimulus of consumer citizenship behavior during the interactions, which supports the social identity theory. What’s more, this paper generalized two antecedents of community identification, namely self-identity and perceived social support. Accordingly, two styles of participation motivations had been found to moderate the above mechanisms. The symbolic motivation of consumers will enhance the effect of the interactions on their self-identities, while utilitarian motivation of consumers will strengthen the relationship between their interactions and perceived social support. From a practical perspective, our findings suggest that activating the identification of community can help stimulate consumers’ citizenship behaviors. To be specific, the online travel communities can enhance consumers’ identification with community in several ways. For example, community can forecast members’ participation motivations through their usage records. For consumers with symbolic motivation, community can provide special platforms or incentives for them to express their self-images so that to enhance their self-identities (Shukla and Rosendo-Rios, 2021; Iloranta and Komppula, 2022). For consumers with utilitarian motivation, community can create convenient pathways for them to seek for the functional value, such as recommending related posts or topics for them to participant. We suggest that such measures, by potentially inducing consumers’ identifications of community, can benefit the online travel communities as a whole. In conclusion, we deeper the understanding of the value co-creation of online communities, their antecedents, and related mechanism.

We first review literature on social exchange theory and online self-presentation, value co-creation of online community, and its manifestation-consumer citizenship behaviors. Thereafter, we use structural equation model to test our proposed conceptual framework. We examine self-identity, perceived social support, identification of community as three antecedents, and the moderate effect of participation motivation. We conclude by discussing theoretical and practical contributions, as well as the implications for future research.

## Literature review and hypotheses

### Social exchange theory and online self-presentation

Social exchange theory proposed that social behaviors of individuals were similar to the commodity exchange during the interactions (Homans, 1958). Blau (2017) expanded the exchange structure from inter-individuals to individual groups. People will continue the interaction if they can get benefits, while they will cease it when its cost was higher (Organ and Konovsky, 1989). Therefore, this theory can explain the counterbalance of costs and benefits for online social activities, especially for those with utilitarian motivation (Liu et al., 2016). In the online travel communities, consumers will probably consider the benefits from the interactions against their costs, which affect their attitudes and subsequent social behaviors toward the members and communities. However, this theory has failed to explain those social behaviors without explicit benefits for consumer. Therefore, we should consider the implicit factors to stimulate the interaction.

Online self-presentation refers to the strategies individuals used to present themselves on personal web pages or social media platform (Tussyadiah and Park, 2018). People will apply the branding principles originating from product marketing to the generation of their ideal self-image online to achieve their goals (Schwabel, 2009). Personal branding means the process individuals use to differentiate themselves from others through their unique value propositions and keep this image consistent across various communication environments (Schwabel, 2009). Research on personal branding initially focused on the public figures in the industry of politic or entertainment (Stanyer, 2008; Marshall, 2010). With the rise of social media, more attention had paid to everyday people who simply present themselves online for seeking pleasure, building friendship, or expressing themselves (Shepherd, 2005; Labrecque et al., 2011; Chen, 2013). Given the virtual nature of technology-mediated communication, consumer can selectively convey the information about themselves (Toma et al., 2008). That is to say, online self-identity is malleable and censored by individual intentionally (Tussyadiah and Park, 2018). Message delivers would present themselves optimistically for catering to message receivers (Tussyadiah and Park, 2018). However, previous researches mainly focus on the antecedents of self-presentation and restricted the context to the self-branding on social media. The influence of self-presentation on consumer behavior needs more attention. In addition, the probability of offline interactions for tourists requires consumers of the online travel communities to balance their positive and honest self-presentation, which had not been fully explored.

### Value co-creation in online community

Customers can solely rely on the information provided by enterprises to make purchase decisions for the traditional product or service. Fortunately, Vargo and Lusch (2004) proposed that products should be customer-oriented instead of enterprise-oriented, and the value of corporate should be created by both customers and enterprises. More specifically, the value co-creation refers to the value of corporation that was jointly created by enterprise, customers, and other stakeholders through the interaction of integrating and applying resources (Vargo and Lusch, 2008a). It exploited the service-dominant instead of product-dominant logic (Vargo and Lusch, 2008b). Ballantyne and Aitken (2007) also mentioned that the brand value depended on not only the unilateral marketing of enterprises, but also the long-term interaction between stakeholders such as enterprises, customers, suppliers, corporate employees, and other online vendors. Furthermore, the synergistic effects of service-dominant logic and value co-creation have proved effective for the brand marketing (Merz et al., 2009). To improve the brand value, the value co-creation between enterprise and customer had been applied to many fields. For example, customers were invited to engage into the designing or manufacturing process in the production field (Zwass, 2010). The customer–customer interaction was as critical as the customer–enterprise interaction in the consuming (Dong et al., 2008).

With the development of Internet, online brand community enabled customers to obtain the product and service experience by browsing the evaluation of other customers, or interacting with the members of community. The role of consumers has turned from passive to active participants. To be specific, customers not only purchase the product, but also participate in the research and development (R&D) through feedback on the online community. They shared their knowledge, skills, and experience to enhance their satisfaction with the purchase journey (Hartley, 2004; Sheth and Uslay, 2007). Moreover, the social well-being (including well-being of consumers, enterprises, and employees) had been enhanced with the help of the members’ interaction on the online travel communities, contributing to the value co-creation (Xue et al., 2021). The value co-creation behavior of consumer was measured by two dimensions, namely intra-role behavior and extra-role behavior (Yi et al., 2013). Intra-role behaviors include the collection of product information, information sharing with other customers, responsible consumption, and the communication. On the other side, extra-role behaviors were represented by feedback, advocacy, helping, and tolerance for service failure (Yi et al., 2013). For the online community, members’ citizenship behaviors have evolved into recommendation, community interaction, community guideline maintenance, helping, and information feedback (Liao, 2021). Existing literature regarded the consumer participation as the value co-creation (Hsieh et al., 2022). However, the intra-role consumer participation such as information searching and problem consulting can contribute to the survival of communities but not bring about the substantial creation of value. Only the extra-role consumer participation can create the competitive value for the communities. Based on the existing research, this study focuses the consumer value co-creation on their extra-role consumer participation, which will be of practical meaning.

### Consumer citizenship behavior

There is a difference between consumer participation and consumer citizenship behavior. Consumer participation on digital platforms can be divided into two forms, namely consumer-to-consumer interaction and consumer-to-brand interaction (Cheung et al., 2021). The former means the sharing of information about the products and brands among consumers, while the latter refers to the contribution of ideas to brands from consumers (France et al., 2015; Tajvidi et al., 2017). Even though consumer participation may facilitate the desirable relationship between consumer and brand, it does not necessarily create value for the business. In this vein, Hollebeek (2011) proposed consumer-brand engagement, which means the level of consumers’ investment from cognition, emotion, and behavior during the specific brand interactions. Consumer-brand engagement can be regarded as the result of intensive consumer participation (Cheung et al., 2021). Indeed, consumer participation or even engagement can contribute to the maintenance of brand community. However, most of the researches focused on consumer engagement with brands or products, whose relationship of interacting parties was transaction-oriented. Research about consumer engagement with other actors such as goal pursuit of traveling in the online travel communities was not enough. Unlike the platform of social-commercial exchange, where the social relationship between participants was not expected to endure, interactions of the online travel community occurred among like-minded peers (Tussyadiah and Park, 2018). Consumer-to-consumer interaction weights more than the traditional consumer-to-brand interaction in such communities; thus, consumer citizenship behavior plays a much more crucial role compared to other online brand communities.

Consumer citizenship behavior originated from organizational citizenship behavior (Yi and Gong, 2008). It was neither necessary for the production or service, nor rewarded by the company, but acted voluntarily and spontaneously by consumer, which benefits the organization (Groth, 2005; Curth et al., 2014; Zhu et al., 2016). Customer citizenship behavior is the result of relationship-oriented rather than transaction-oriented business (Groth, 2005). Consumer citizenship behavior represents the sound relationship between consumers and enterprises. On the contrary, consumer tends to pay out less in a simple transaction relationship. Consumers are not only the user of services and products, but also the leader of value creation (Heinonen et al., 2013). On the one hand, the active participation of consumer can save the service cost and make the transmission of value smoother. On the other hand, the other consumer can have a better experience with the help of consumer citizenship behavior (Rosenbaum and Massiah, 2007; Vargo and Lusch, 2008b). Given the benefits of consumer citizenship behavior, scholars have explored its contributors based on different theories. According to the theory of emotional initiation, people are more likely to behave prosocially when they are in a positive emotion (Romani et al., 2013). However, emotional priming is often temporary; this short-term emotional state cannot explain the long-term behavior such as consumer citizenship behavior. Based on the social exchange theory, consumers have the responsibility to conduct a reciprocal citizenship behavior after obtaining the value shared by the enterprise or others; otherwise, they will feel the social pressure or guilt (Anaza and Zhao, 2013), but this theory cannot explain the situation that what the consumer pays out was much more than what they can receive. There was other explanation that consumer citizenship behavior was stimulated by the empathy (Batson et al., 2002; Bove et al., 2009). Nonetheless, the effect of empathy was not stable and cannot be significant for other studies (Kruger, 2003). Therefore, the mechanism of consumer citizenship needs to be explored further. What’s more, the majority of studies in tourism and hospitality industries focuses on employee citizenship behavior (Chen, 2016), and more attention should be paid to the consumer citizenship behavior, especially for the online travel communities.

### Consumer citizenship behavior mechanism

Interaction can deeper consumer’s understanding of the community (e.g., behavior norms, unique culture, and significance), so that they can expand and express their self-identity (Watts and Dodds, 2007). In the meantime, consumers take delight in sharing their knowledge of the community and their experience of the travel (Muniz and Hamer, 2001). Consumers can not only gain recognition and respect from other members in the community, but also feel a sense of achievement and superiority in the process of sharing (e.g., product information, personal feelings after travel, and information of outdoor travel; Nambisan and Baron, 2009). Consumer interaction helps members obtain and maintain the special identity and status of the community, so that further realize their self-worth, self-improvement, and self-identity (Yen et al., 2011).

Consumer self-identity has a direct impact on their identification of community (Schau and Muniz, 2002). Consumers construct, define, and improve their self-image through interaction (e.g., share their travel experience). Moreover, the more consistency between consumer self-image and the community-image, the more affinity consumers show to the community, and the more they identify with it (Hogg et al., 2000; Belén del Río et al., 2001). Consumers are likely to identify with the community when the members of it share common interests, lifestyles, consumption habits, and favorite fields (Muñiz and O’Guinn, 2001). Consumers use their creativity and expression to coordinate the boundaries between self-identity and community members in the interaction. Furthermore, they form and maintain their community membership, emphasizing the similarities with other community members and the differences with non-members (Schau and Muniz, 2002). Meanwhile, consumers poured their emotions into the community during the interaction (e.g., actively participate in communication and sharing), such that enhancing their identifications with the community (Mcalexander et al., 2002; Algesheimer et al., 2005).

According to the social exchange theory, consumer wants to be reciprocal after getting a sense of achievement, sense of superiority, self-esteem, and self-enhancement (Dholakia et al., 2004; Nambisan and Baron, 2009; Kuo and Feng, 2013). They would like to make contributions to the community to reduce the sense of guilt (Blau, 2017). In the construction of community, consumer self-identity may directly increase their identification of the community and thus obviously promote their citizenship behaviors (Schau and Muniz, 2002). Based on the discussion, we propose the following hypothesis:

**Hypothesis 1a.** Consumer interaction exerts its significant and positive impact on self-identity.

**Hypothesis 1b.** Self-identity exerts its significant and positive impact on the community identification.

**Hypothesis 1c.** Self-identity exerts its significant and positive impact on consumer citizenship behavior.

Perceived social support is defined as the perception or experience of being loved, cared for, respected, and valued (Zimet et al., 1988; Mana et al., 2021). It is a subjective feeling of being part of a social network of mutual assistance (Zimet et al., 1988; Mana et al., 2021). Social support is generally divided into objective support and subjective support (Yu et al., 2015). Objective support is visual or actual existence such as material support and network support, and it does not shift from individual feelings; subjective support is interpersonal emotional support such as intimate interaction and self-esteem satisfying, and it occurs when individuals are respected and understood in social life (Yu et al., 2015). The asymmetry of the information increases the uncertainty and risk for purchase; thus, consumers will ask for help in the community (Kunz and Seshadri, 2015). During the interaction, consumers obtain objective support from the community, maintain a mutual assistance relationship with other members, and enhance their sense of social support (Xue et al., 2021).

These supports enable consumers to have a further understanding of the community products and culture. In the meantime, consumers realize that the community can not only provide a platform for them to show their personality and lifestyle, but also meet the members who shared the similar values with them, thus increasing their identification of the community (Keller, 2001).

Furthermore, perceived social support can improve individuals’ quality of life, increase their subjective well-being, and thus promote their positive behaviors (Yao et al., 2015). To be specific, consumer will provide more help and concern to other members in the community when they perceived social support from the interaction. According to the social exchange theory, consumer wants to pay back after they received the social support both objectively and subjectively (Eisenberger et al., 2002; Crocker and Canevello, 2008). They are more likely to transfer from beneficiaries to contributors (Shakespeare-Finch and Obst, 2011). Consumers will voluntarily help the community promote and give feedback on related issues out of gratitude.

Based on the discussion, we propose the following hypothesis:

**Hypothesis 2a.** Consumer interaction exerts its significant and positive impact on perceived social support.

**Hypothesis 2b.** Perceived social support exerts its significant and positive impact on the identification of community.

**Hypothesis 2c.** Perceived social support exerts its significant and positive impact on consumer citizenship behavior.

Consumers with community identification comply with the behavioral norm, rituals, and goals of the community, and they will strive for the welfare of it (Bhattacharya et al., 1995). Community identification can enhance consumer’s understanding of the community culture (Watts and Dodds, 2007), consumer’s loyalty to the community (Dessart and Veloutsou, 2021), and their active participation (Algesheimer et al., 2005). For example, consumer who identifies with the community will be more willing to recommend the community to the out-group, support the development of the community, and conduct the citizenship behaviors (Hsu et al., 2015; Deng et al., 2021). Research also found that the identification of community can strengthen the loyalty of inactive consumers and stimulate them to provide help for the benefits of the community (Dessart and Veloutsou, 2021). Based on the discussion, we propose the following hypothesis:

**Hypothesis 3.** The community identification exerts its significant and positive impact on the consumer citizenship behavior.

### The moderate effect of different participation motivation

Different participation motivations will lead to different psychological processes for consumers. The symbolic motivation refers to the desire for social status and prestige (Kastanakis and Balabanis, 2014). It is generally represented by the consumption embodying personal taste, personality, status, and identity (Shukla and Rosendo-Rios, 2021). Since tourism was regarded as “luxury consumption” to some of the population, consumer with symbolic motivation wants to express their personality, social status, and self-image through the interaction in the online travel community. They focus more on their social status rather than the functional value (Shukla and Rosendo-Rios, 2021; Iloranta and Komppula, 2022). Therefore, symbolic motivation enhances the relationship between interaction and self-identity.

On the contrary, utilitarian motivation means that the driver of the action depends on whether the task or goal is achieved effectively (Akram et al., 2021). People with utilitarian motivation care more about the functional value provided by the interaction. That is, consumer with this incentive will seek for the content which is appropriate for their purpose from the community (Dabbous and Barakat, 2020; Akram et al., 2021). For example, they are concerned about information of the tourism products and ask for the social support from the interaction. Therefore, utilitarian motivation enhances the relationship between interaction and perceived social support.

Based on the discussion, we propose the following hypothesis:

**Hypothesis 4a.** Symbolic motivation positively moderates the relationship between consumer interaction and self-identity.

**Hypothesis 4b.** Utilitarian motivation positively moderates the relationship between consumer interaction and perceived social support.

**FIGURE 1:**
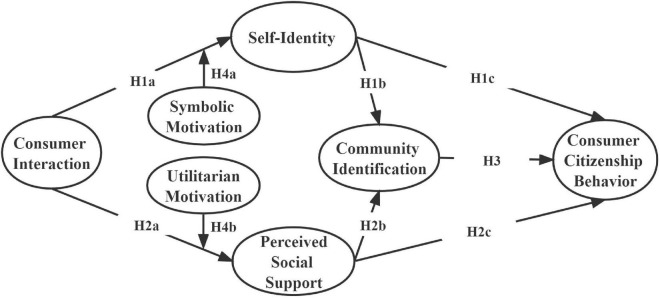
Conceptual model.

In summary, Figure 1 shows the research model proposed in this study.

## Methodology

### Survey instrument

A survey questionnaire was developed to collect data so that the hypotheses can be validated. The questionnaire included four parts. The first part was a brief introduction. We introduce the purpose of the survey to the respondents, saying it was to promote the development of the online travel community. The second part was the scenario imagining. The participants were told to imagine they were surfing the online travel community as usual. To stimulate the different motivations of consumers, participants in the group of utilitarian motivation were told that they were participating in the online travel community just for information searching, while participants in the group of symbolic motivation were asked to imagine that they were participating in the online travel community not for information searching but for sharing of their extraordinary and memorable travel experience which can bring them social status. The third part was the scale of each construct. The fourth part is the item related to the demographic information of the participants.

The scale items were adapted from the extant research. Scale items for consumer interaction were adapted from Cao et al. (2021), for example, “I always actively take part in community discussions and have close and intensive interactions with other members of the online travel community.” Scale items for self-identity were adapted from Confente et al. (2020), for example, “I would feel totally satisfied with myself if I interact with other members in the community.” Scale items for perceived social support were adapted from Zhu et al. (2016), for example, “Some people expressed interest and concern in my well-being of the travel.” Scale items for the community identification were adapted from Algesheimer et al. (2005), for example, “I see myself as a part of the community.” Scale items for consumer citizenship behavior were adapted from Groth (2005), for example, “Recommend the community to people interested in the community’s products/services.” All scale items were measured by a seven-point Likert scale, where 1 represents “strongly disagree” and 7 represents “strongly agree.”

### Data collection

We selected some famous online travel community in China (e.g., Tripadvisor; Iqingyi.com; Qyer.com; Mafengwo.com) as the study context. The questionnaire was generated by the Sojump.com (one of the biggest platforms for survey in China). The data were collected from January 2022 to February 2022. All participants signed a written consent form before the survey and were paid for their participation.

Some questions were set for the screening before the survey, such as the following: (1) Do you participate in the online community? (2) Will you go to the online travel community? (3) What is the name of the online travel community you prefer to participate in? (4) Will you frequently visit your preferred online travel community? Only respondents who answered “yes” to the above questions (1, 2, and 4) or gave an appropriate name for question 3 were considered for the further participation of our survey. Six hundred (600) respondents were selected for the final survey, but seven respondents were excluded for their failure of the attention test. A total of 593 respondents were retained for the further analysis.

### Demographic details of samples

The demographic information of participants (see Table 1) revealed that 308 respondents were male (51.94%), while 285 respondents were female (48.06%). In addition, the majority concentrate between 26 and 35 ages (52.95%), holding junior college or undergraduate degree (86.17%), and with an income between 5,001 and 8,000 RMB per month (39.12%).

**TABLE 1:** Respondents’ demographic information (*n* = 593).

Categories		Frequency	Percentage (%)
**Gender**			
	Male	308	51.94%
	Female	285	48.06%
**Age**			
	Below 18	4	0.67%
	18–25	180	30.35%
	26–35	314	52.95%
	36–45	75	12.65%
	46–60	18	3.04%
	Above 60	2	0.34%
**Education**			
	High school or below	25	4.22%
	Junior college or undergraduate	511	86.17%
	Master or above	57	9.61%
**Income (RMB)**			
	Less than 5,000	108	18.21%
	5,001–8,000	232	39.12%
	8,001–17,000	181	30.52%
	17,001–25,000	51	8.60%
	Above 25,000	21	3.54%

## Results

### Common method bias

All the questionnaires were answered anonymously, which can relieve the psychological stress and common method bias of participants (Podsakoff et al., 2003). A prior procedural remedy was conducted during the pretest to refine the scale items so that it can avoid potential ambiguities (Podsakoff et al., 2012). There were three questions to keep the attention of the respondents during their reading. Moreover, the common method variance (CMV) was analyzed by Harman’s one-factor test (Podsakoff et al., 2003). The result of this test showed that the total variance was less than 50%, which means that common method bias was not a concern in the study according to the guideline of Podsakoff et al. (2003). Furthermore, the path coefficients and correlations of the constructs for the assessment of the structural model had various degrees of significance, which showed the result was not confounded by common method bias (Alkhalifah, 2021).

### Evaluation of measurement model

Although the items of the questionnaire are from mature scales, their applicability still needs to be tested. A confirmatory factor analysis was performed using the AMOS 22.0 software to facilitate further validation of the internal structural validity of the measurement scale. The standardized factor loadings were greater than 0.50 (see Table 2), which were considered significant according to the two-step approach of structural equation modeling (Anderson and Gerbing, 1988).

**TABLE 2:** Measure items, the reliability, and convergent validity.

Items	Standardized factor loading	CR	α	AVE
**Consumer Interaction (CI)**		**0.922**	**0.921**	**0.702**
My community interaction contained large amount of information about the outdoor travel.	0.807			
I share my knowledge of outdoor travels with others in the community.	0.846			
I always post new threads in the community and will get response quickly from others.	0.867			
I always actively take part in community discussions and have close and intensive interactions with other members of the online travel community.	0.864			
I always participate in two-way communications for sharing experiences and feeling.	0.802			
**Self-Identity (SI)**		**0.917**	**0.917**	**0.733**
I think of myself as someone who is concerned about the outdoor travel.	0.847			
I think of myself as a travel enthusiast.	0.849			
Interacting with other members in the community make me feel like a travel enthusiast.	0.885			
I would feel totally satisfied with myself if I interact with other members in the community.	0.843			
**Perceived social support (SS)**		**0.902**	**0.902**	**0.698**
Some people offered me suggestions to solve the problem.	0.814			
Some people helped me discover the destination and provided me with related knowledge.	0.853			
Some people comforted and encouraged me to pursue my ideal travel.	0.863			
Some people expressed interest and concern in my well-being of the travel.	0.810			
**Community identification (CID)**		**0.958**	**0.957**	**0.693**
I am very attached to this online travel community.	0.822			
Other members of this online travel community share the same objectives as me.	0.814			
The friendships I have with other members of this online travel community mean a lot to me.	0.824			
If the members of this online travel community planned something, I’d think of it as something “we” would do rather than something “they” would do.	0.847			
I see myself as a part of the online travel community.	0.833			
**Consumer Citizenship Behavior (CB)**		**0.956**	**0.956**	**0.667**
**Customer citizenship behavior: recommendations**				
Refer fellow students or coworkers to the community.	0.801			
Recommend the community to your family.	0.824			
Recommend the community to your peers.	0.831			
Recommend the community to people interested in the community’s products/services.	0.821			
**Customer citizenship behavior: helping customers**				
Assist other customers in finding tourism products.	0.831			
Help others with their selection of traveling route.	0.857			
Teach someone how to use the service of the community correctly.	0.842			
Explain to other customers how to use the service of the community correctly.	0.821			
**Customer citizenship behavior: providing feedback**				
Fill out a customer satisfaction survey.	0.783			
Provide helpful feedback as to how this online travel community can be improved.	0.777			
Provide information when surveyed by this online travel community.	0.788			

α, Cronbach’s alpha; CR, composite reliability; AVE, average variance extracted.

The results of the validation factor analysis showed that the fit of the model met the standard. To be specific, normal chi-square/degrees of freedom (χ^2^/df) = 2.549, relative fit index (RFI) = 0.920, comparative fit index (CFI) = 0.954, Tukey–Lewis index (TLI) = 0.950, normed fit index (NFI) = 0.927, incremental fit index (IFI) = 0.954, root mean square error of approximation (RMSEA) = 0.051. Therefore, the fitting index of the model meets the statistical requirements, so the validity of the data was acceptable (Alkhalifah, 2021). Furthermore, Cronbach’s alpha (α) values were greater than the threshold of 0.70 (Anderson and Gerbing, 1988), demonstrating a sound internal consistency among the items of the constructs and the reliability of the model. The findings of composite reliability (CR) of the model were greater than the threshold of 0.60 (Bagozzi and Yi, 1988), manifesting a fine internal reliability. The discriminant validity was assessed by the average variance extracted (AVE). The results (see Table 2) showed the internal consistency (i.e., α, CR, and AVE) of the model. In addition, all the square roots of AVEs had been shown to exceed the coefficients between each pair of constructs (see Table 3), which indicated the scale had a good discriminative validity.

**TABLE 3:** Construct correlation and square roots of AVE values.

	CI	SI	SS	CID	CB
CI	**0.838**				
SI	0.507	**0.856**			
SS	0.42	0.466	**0.835**		
CID	0.499	0.563	0.438	**0.832**	
CB	0.639	0.552	0.555	0.475	**0.817**

CI, consumer interaction; SI, self-identity; SS, perceived social support; CID, community identification; CB, citizenship behavior. The bold numbers are the square roots of the AVE values.

### Evaluation of structural model

In the second step, the hypotheses H1 (i.e., H1a, H1b, H1c), H2 (i.e., H2a, H2b, H2c), and H3 had been tested. The result showed that the model fit well (e.g., χ^2^ = 1387.994, df = 520, χ^2/^df = 2.669, RFI = 0.917, CFI = 0.950, TLI = 0.946, NFI = 0.923, IFI = 0.950, and RMSEA = 0.053).

Pathway analysis of the structural equation showed that consumer interaction had a significant and positive effect on consumer self-identity (β = 0.580, *t* = 13.142, *p* < 0.001); thus, H1a was supported; consumer self-identity had a significant and positive effect on consumer community identification (β = 0.415, *t* = 9.127, *p* < 0.001); thus, H1b was supported; consumer self-identity had a significant and positive effect on consumer citizenship behavior (β = 0.225, *t* = 5.552, *p* < 0.001); thus, H1c was supported; consumer interaction had a significant and positive effect on consumer perceived social support (β = 0.459, *t* = 10.150, *p* < 0.001); thus, H2a was supported; consumer perceived social support had a significant and positive effect on consumer community identification (β = 0.253, *t* = 5.743, *p* < 0.001); thus, H2b was supported; consumer perceived social support had a significant and positive effect on consumer citizenship behavior (β = 0.297, *t* = 7.476, *p* < 0.001); thus, H2c was supported; consumer community identification had a significant and positive effect on consumer citizenship behavior (β = 0.408, *t* = 9.804, *p* < 0.001); thus, H3 was supported. The above results are summarized in Table 4.

**TABLE 4:** Hypothesis testing results.

Hypotheses	Path	Proposed effect	Path coefficient	*P* values	Results
H1a	CI-SI	Positive	0.58	< 0.001	Supported
H1b	SI-CID	Positive	0.415	< 0.001	Supported
H1c	SI-CB	Positive	0.225	< 0.001	Supported
H2a	CI-SS	Positive	0.459	< 0.001	Supported
H2b	SS-CID	Positive	0.253	< 0.001	Supported
H2c	SS-CB	Positive	0.297	< 0.001	Supported
H3	CID-CB	Positive	0.408	< 0.001	Supported

### Moderation test

In addition to the model, we added two more groups to the original sample data in the Amos software, namely “utilitarian motivation” and “symbolic motivation.” Then, we divided the data into two groups according to the answer of the first question in the “File Name.” The group answered “1” belongs to “utilitarian motivation,” while the group answered “2” belongs to “symbolic motivation.” After that, the qualified model was set in “Manage Model” and limit the regression coefficients of “utilitarian motivation” and “symbolic motivation” to be equal, for example, UM30 = SM30; UM31 = SM31; UM32 = SM32; UM33 = SM33; UM34 = SM34; UM35 = SM35; UM36 = SM36. Specifically, UM represents the regression coefficient of the group “utilitarian motivation,” while SM represents the regression coefficient of the group “symbolic motivation.” Finally, structural equation model analysis was conducted.

**FIGURE 2:**
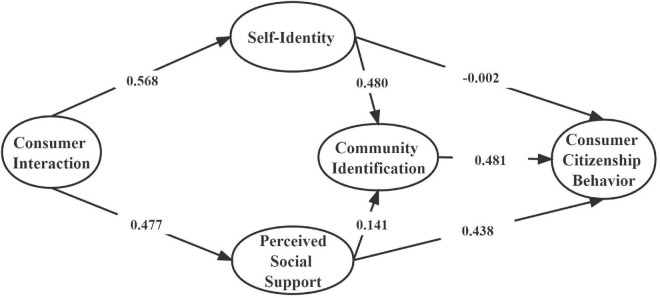
Standardization path map of unlimited model of utilitarian motivation. χ^2^ = 3755.774, df = 1696, χ^2/^df = 2.214, TCL = 0.941, CFI = 0.941, NFI = 0.897, RMSEA = 0.032.

**FIGURE 3:**
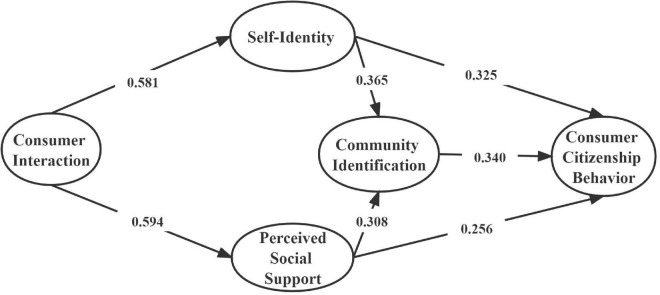
Standardization path map of unlimited model of symbolic motivation. χ^2^ = 3755.774, df = 1696, χ^2/^df = 2.214, TCL = 0.941, CFI = 0.941, NFI = 0.897, RMSEA = 0.032.

**FIGURE 4:**
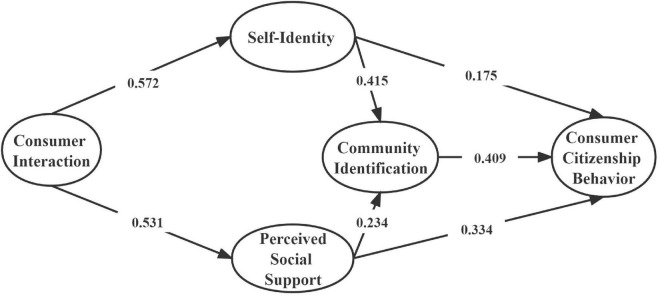
Standardization path map of limited model of utilitarian/symbolic motivation. χ^2^ = 3774.532, df = 1703, χ^2/^df = 2.216, TCL = 0.941, CFI = 0.941, NFI = 0.897, RMSEA = 0.032. When standardized path coefficients were set as equal, the path maps were the same for both utilitarian and symbolic motivation.

According to the standardized path map of the above comparison (see Figures 2, 3, 4), we can find that the chi-square values and degrees of freedom vary in both the restricted and unrestricted models, but there is no significant change in some fitting metric. For further testing, we combined the final output of the text to determine whether there is a significant difference between the restricted model and the non-restricted model. The specific analysis is shown in the following Table 5. The results showed that the change of “the change amount/the change of degree of freedom” (△χ^2^/△df = 18.758/7, *p* < 0.01) was significant after limiting all the coefficients of the structural equations to be equal. Therefore, it could be judged by the chi-square value that the regulatory effect of the type of participation motivation was significant.

**TABLE 5:** Unlimited model (free estimates for all parameters).

Model	df	χ ^2^	*p*	NFI Delta-1	IFI Delta-2	RFI rho-1	TLI rho-2
Model Number 2	7	18.758	0.009	0.001	0.001	0.000	0.000

The SPSS software was used to further test the regulatory effects of the type of participation motivation. Independent sample *t*-test was performed, and the result revealed that perceived social support of consumers with utilitarian motivation was significantly stronger than those with symbolic motivation (M_utilitarian_ = 5.540, M_symbolic_ = 5.005, *t* = 9.584, *p* < 0.001); self-identity of consumer with symbolic motivation was significantly stronger than those with utilitarian motivation (M_utilitarian_ = 4.803, M_symbolic_ = 5.426, t = −10.289, *p* < 0.001). Therefore, H4a and H4b were supported. The above results are summarized in Table 6.

**TABLE 6:** Summary of hypothesis testing results.

Hypotheses	Proposed effect	Results
H1a	Consumer interaction exerts its significant and positive impact on self-identity.	Supported
H1b	Self-identity exerts its significant and positive impact on community identification.	Supported
H1c	Self-identity exerts its significant and positive impact on consumer citizenship behavior.	Supported
H2a	Consumer interaction exerts its significant and positive impact on perceived social support.	Supported
H2b	Perceived social support exerts its significant and positive impact on community identification.	Supported
H2c	Perceived social support exerts its significant and positive impact on consumer citizenship behavior.	Supported
H3	Community identification exerts its significant and positive impact on the consumer citizenship behavior.	Supported
H4a	Symbolic motivation positively moderates the relationship between consumer interaction and self-identity.	Supported
H4b	Utilitarian motivation positively moderates the relationship between consumer interaction and perceived social support.	Supported

## Discussion

Our study investigated consumer citizenship behavior in the online travel communities. The findings strengthen the research of the study. First, consumer interaction exerts its effect on two types of psychological states, namely self-identity and perceived social support. Second, these two types of psychological states are two important antecedents of consumer citizenship behavior in terms of their direct and indirect impacts. Third, both types of psychological states significantly and positively affect consumer identification of community. Furthermore, consumer identification of community has a significant and positive influence on consumer citizenship behavior. Lastly, different motivations of the community participation can moderate the effect of consumer interaction on the psychological states.

Moreover, consumer self-identity has more influence on their identification of community than their sense of social support, while customer perceived social support has a greater impact on customer citizenship behavior than customer self-identity (see Table 4). This is because consumer not only gains information and knowledge of the tourism product, but also develops a special attachment to the online travel community (Cao et al., 2021). This finding supports the argument that the more social support consumers receive from the online community, the more they will identify with the community members (Matute et al., 2019), which contributes to their positive behaviors in the community. On the contrary, self-identity is a considerable factor of community identification. This finding can reinforce the viewpoint that self-brand similarity together with the community participation in identity-salient context contributes to consumers’ brand identity (Liao et al., 2021). However, since self-identity growing out of self-presentation enables consumer to focus more on managing their desired images to others (Goffman, 1959), consumer may pay less attention to the others and show less altruistic behavior. Therefore, community should adopt invention to enhance consumer’s citizenship behavior through the path of self-identity, such as priming them with the social identity.

This study also considered the moderating effect of participation motivation of consumers on the relationship between their interactions and their psychological states. More precisely, consumer with symbolic motivation would be more willing to present or extend their ideal image on the online travel communities and thus strengthen their self-identity through the interactions (Goffman, 1959; Shukla and Rosendo-Rios, 2021). Meanwhile, consumers with utilitarian motivation tended to seek for the information and knowledge from the online travel communities. During this process, consumers not only obtain the functional support but also the emotional support from the communities’ members (Xue et al., 2021). Therefore, their motivations will enhance the effects of their interactions on their sense of social support (Dabbous and Barakat, 2020; Akram et al., 2021).

### Theoretical implications

This study provides some theoretical implications. First, our paper discusses the influence of online community interactions on consumers’ citizenship behaviors, enriching the relevant researches of consumer citizenship behaviors. Previous studies of consumer citizenship behaviors mainly explored their antecedents from the perspective of enterprises, such as corporate social responsibility (CSR; Abdelmoety et al., 2022), cause-related marketing strategy (Deng et al., 2021), service climate (Qiu et al., 2021), and recovery justice of service failure (Zhu et al., 2021). This school of research regarded consumers as outsiders rather than the cooperator of the company. Our findings provide another perspective from consumers, who can conduct citizenship behaviors intrinsically instead of extrinsically. Not only the external incentives (e.g., reputation of the company), but also the internal factors of consumers (e.g., participation motivation) should be considered for the community value co-creation.

Second, this study distinguished consumer citizenship behavior from consumer’s participation. At present, the majority of scholars focus on consumer behaviors such as the consumer loyalty and consumer participation (Cheung et al., 2021). However, these behaviors cannot be equal to the value co-creation of communities. Moreover, overcoming social loafing of online community can lead to consumer interaction, but not the value co-creation (Lv et al., 2021). According to the theory of the value co-creation, the sustainable development of online community largely depends on consumer citizenship behavior, instead of the passive participation in the community (Liao, 2021). Therefore, how to stimulate customer citizenship behavior is particularly important. Given this reason, this paper chooses community interaction as the antecedent to explore its effects on consumer citizenship behavior, which can deeper understanding of the value co-creation of online community.

Third, this study expands the research scope of the social identity theory of online community. Even though previous literature mentioned that the identification of community was the driver of consumer participation of the online travel communities, they had not detailed mechanisms of community identification (Liao et al., 2021). This study provides two antecedents of community identification, namely self-identity and perceived social support. What’s more, different style of motivation has been found to affect the above mechanism. To be specific, symbolic motivation can positively moderate the relationship between consumer interaction and self-identity, while utilitarian motivation can positively moderate the effects of consumer interaction on perceived social support. The former findings apply the self-presentation theory from actual self to the virtual self in online community (Goffman, 1959); the latter findings extend the social exchange theory from material level to spiritual level. When consumers receive the functional support from the online travel communities, they will not only show gratitude to the members of communities but also attach more attachments and identification to the community.

### Managerial implications

This work also has critical implications for practice. Consumers can express themselves and obtain functional support and friendship from community interaction, which can enhance their self-identity and perceived social support. Furthermore, these two psychological states can stimulate their citizenship behaviors directly or indirectly through the identification of community. Therefore, enterprises can initiate more activities to stimulate consumer interactions (e.g., discounts for group purchase, voting for the favorite members, and registration of offline travels). Consumer interactions not only enhance their participation, but also their emotional connection and identity to the community. To be specific, communities can adopt “push and pull” methods to achieve the value co-creation. On the one hand, the community can create a convenient button for consumers to respond timely regards to their product experience and evaluation of the business, so as to help business improve their products. On the other hand, business can stimulate consumer interactions through external incentive, which encourages members to publish travel notes, spread positive word of mouth, and be the stakeholders of the community.

What’s more, communities should provide specific help to consumers with different participation motivations. For consumer with utilitarian motivation, business can recommend related posts or topics for them to participate. In the meantime, experienced members can be invited to interact with the new comers for the specific issue. For consumers with symbolic motivation, business can create more opportunities for them to express themselves (e.g., special cultural event and regular voting for the popular post) and reward them with the premium titles. It is also worth providing this group of consumers with sense of superiority and accomplishment from the community interaction (Shukla and Rosendo-Rios, 2021; Iloranta and Komppula, 2022).

Lastly, business can try to use multi-sensory strategy to stimulate consumer interaction (Lv et al., 2020). For example, community can adapt to different consumers with different visual and aural interaction way. To be specific, interaction design can be in warm color and match with supportive music to enhance consumers’ sense of social support, while it can be in cold color and decorated with classical music to highlight the personality and status for those who care more for self-identity.

### Limitations and directions for future research

There are several limitations of this research. First, the ecological validity of the research can be improved. Since tourism products are experiential and hedonistic products, consumers are more willing to invest time and energy into the searching of relevant information. Therefore, the members of the online travel community may interact more frequently than other brand communities. Whether this mechanism can apply to other communities (e.g., the search or utilitarian products’ communities) needs further validation in future. Second, there are potential risks for endogenous problems in the model. As a dependent variable, consumer citizenship behavior may be counteractive at consumer interaction. That is, the more consumer citizenship behavior, the more consumer interaction. Therefore, future studies can examine whether customer citizenship behavior has a positive effect on consumer interactions in online communities. Third, consumer personality has not been considered in this study. Consumers with different personalities may have differences in their cognition, affection, and responses to the community interactions. Therefore, further research can explore the effects of individual’s personalities on consumer interaction. Lastly, the value co-creation model constructed in this paper is based on intra-community interactions. However, there are also extra-community interactions, whereby the frequency and quality of the interaction may be quite different. Therefore, future studies can explore the synergy of both intra- and extra-community interactions.

## Data availability statement

The original contributions presented in the study are included in the article/supplementary material, further inquiries can be directed to the corresponding author/s.

## Ethics statement

The studies involving human participants were reviewed and approved by Jinan University. The patients/participants provided their written informed consent to participate in this study.

## Author contributions

BG, HC, YL, and RL conceived and designed experiments. YL, RL, and AW carried out the experiments and analyzed the experimental results. BG wrote the manuscript. AW edited the manuscript. All authors contributed to the article and approved the submitted version.
